# Efficacy of ixekizumab in patients with moderate-to-severe plaque psoriasis and prediabetes or type 2 diabetes

**DOI:** 10.3389/fmed.2022.1092688

**Published:** 2023-01-27

**Authors:** Alexander Egeberg, Joseph F. Merola, Knut Schäkel, Luis Puig, Patrick D. Mahar, Isabella Yali Wang, Imre Pavo, Christopher Schuster, Christopher E. M. Griffiths

**Affiliations:** ^1^Bispebjerg and Frederiksberg Hospital, Copenhagen University, Copenhagen, Denmark; ^2^Division of Rheumatology, Department of Dermatology and Department of Medicine, Harvard Medical School, Brigham and Women's Hospital, Boston, MA, United States; ^3^Department of Dermatology, University Hospital Heidelberg, Heidelberg, Germany; ^4^Dermatology Department, Hospital de la Santa Creu i Sant Pau, Barcelona, Spain; ^5^Eli Lilly and Company, Indianapolis, IN, United States; ^6^Department of Dermatology, Royal Children's Hospital, Faculty of Medicine, Nursing and Health Sciences, Skin Health Institute, The University of Melbourne, Melbourne, VIC, Australia; ^7^Department of Dermatology, Medical University of Vienna, Vienna, Austria; ^8^Dermatology Centre, Salford Royal Hospital, NIHR Manchester Biomedical Research Centre, University of Manchester, Manchester, United Kingdom

**Keywords:** moderate-to-severe psoriasis, type 2 diabetes, prediabetes, ixekizumab, obesity

## Abstract

**Objective:**

Patients with psoriasis have an increased prevalence of type 2 diabetes when compared to the general population. Research suggests that type 2 diabetes (T2D) as well as obesity may have an impact on patients' response to treatment. This post-hoc analysis reports the efficacy of ixekizumab in treating moderate-to-severe psoriasis in patients with prediabetes or T2D.

**Method and materials:**

UNCOVER-1, UNCOVER-2, and UNCOVER-3 were three Phase 3, multicenter, randomized, double-blind, placebo-controlled trials that evaluated the efficacy and safety of ixekizumab in adult patients with moderate-to-severe psoriasis. Patients were aged ≥18 years with chronic moderate-to-severe psoriasis (defined as ≥10% body surface area affected, static Physician Global Assessment ≥3, and Psoriasis Area and Severity Index [PASI] ≥12 at screening and baseline) who were candidates for phototherapy or systemic therapy. UNCOVER-1, UNCOVER-2, and UNCOVER-3 participants received ixekizumab as per label (that is, an initial dose of two subcutaneous injections [160 mg in total] at Week 0, followed by 80 mg every 2 weeks through Week 12 and 80 mg every 4 weeks thereafter through Week 60).

**Results:**

The proportions of patients with prediabetes, T2D and normoglycemia that achieved PASI75, PASI90, and PASI100 at Week 60 were similar. Results suggest that patients with T2D were slower to achieve PASI100 than patients with prediabetes or those with normoglycemia. Ixekizumab had no effect on any metabolic markers in patients receiving the treatment.

**Conclusions:**

Despite the higher rate of obesity and extreme obesity in patients with prediabetes and T2D, ixekizumab was an efficacious treatment in treating patients with psoriasis.

## Introduction

Plaque psoriasis is a chronic, inflammatory skin disease affecting approximately 1% of adults worldwide (1–3). Patients with psoriasis have a higher risk of developing several metabolic conditions, including type 2 diabetes (T2D), and this is thought to be a result of shared underlying pathophysiologic drivers of both diseases (4). Recent data suggest that a higher prevalence of prediabetes (precursor of T2D defined with glucose levels above normal but below diabetes diagnostic levels) in patients with psoriasis can be assumed too (5). Beyond T2D, patients with psoriasis also have increased rates of other metabolic syndrome components such as obesity, hypertension, and atherogenic dyslipidemia, such as hypertriglyceridemia and reduced high-density lipoprotein (HDL) levels (6, 7).

Recent research suggests that in patients with psoriasis, T2D reduces the response to biologic treatments, especially interleukin-17 inhibitors, after 6 months treatment (4). Additionally, obesity and hypertension may play a role in reducing responses to systemic and biologic treatments for psoriasis (4, 8). Whether this holds true for prediabetes remains unknown.

This study investigates the efficacy of ixekizumab in long-term treatment of moderate-to-severe psoriasis in patients with prediabetes or T2D (9, 10). As there is a significant correlation between psoriasis and diabetes, it is important to understand the efficacy in these patients so that clinicians can make more informed treatment decisions (11).

## Methods

UNCOVER-1 (NCT01474512), UNCOVER-2 (NCT01597245), and UNCOVER-3 (NCT01646177) were Phase 3, multicenter, randomized, double-blind, placebo-controlled studies that evaluated the efficacy and safety of ixekizumab in adult patients with moderate-to-severe psoriasis (9, 10).

This *post-hoc* analysis included patients who received ixekizumab as per label (initial dose of two subcutaneous injections [160 mg in total] at Week 0, followed by 80 mg every 2 weeks through week (W) 12 and 80 mg every 4 weeks thereafter through W60). Patients were grouped based on the T2D diagnosis, presence of prediabetes, or normoglycemia at baseline. T2D was defined as recorded in the patient's medical history and prediabetes as a fasting glucose level ≥5.7 mmol/L (12). Patients with type 1 diabetes mellitus were excluded from the analysis.

Efficacy was assessed by the proportion of patients achieving ≥75% improvement in Psoriasis Area and Severity Index (PASI75), PASI90, and PASI100 from baseline through W60. Patients' body mass index (BMI), blood pressure, and levels of various metabolic parameters were also assessed at multiple timepoints through W60.

Data were analyzed using logistic regression analysis and mixed models with repeated measures analysis. Continuous variables were analyzed using *t-*tests. Non-responder imputation (NRI) was used for missing categorical data.

## Results

### Baseline characteristics

Among the 564 ixekizumab-treated patients with moderate-to-severe psoriasis included in this *post-hoc* analysis, 118 (20.9%) and 40 (7.1%) had prediabetes and T2D, respectively. In 18 (3.2%) patients with unknown T2D, fasting baseline glucose value ≥5.6 mmol/L and also ≥7.0 mmol/L and variable post-baseline values, were included in the prediabetes group. Baseline patient demographics and characteristics are summarized in Table 1. Patients with prediabetes or T2D were older and had numerically higher rates of obesity (particularly extreme obesity [BMI ≥ 40]), hypertension, hyperlipidemia, and fasting serum glucose levels at baseline compared with patients with normoglycemia. Of the patients with T2D, 77.5% had hypertension and 97.5% of patients were above normal weight. Psoriasis severity was comparable across patients with prediabetes, T2D, and normoglycemia; however, psoriasis severity and impact scores (PASI, Body Surface Area, and Dermatology Life Quality Index) were slightly higher in patients with diabetes and prediabetes.

**Table 1:** Baseline patient demographics and characteristics in ixekizumab-treated patients included in this *post-hoc* analysis.

	**T2D (*N =* 40)**	**Prediabetes (*N =* 118)**	**Normoglycemia (*N =* 406)**
**Age, years**	55.6 ± 9.7	50.2 ± 12.5	41.8 ± 12.5
**Gender, male**, ***n*** **(%)**	28 (70.0)	88 (74.6)	263 (64.8)
**Race**, ***n*** **(%)**			
Black or African American	2 (5.0)	1 (0.8)	5 (1.2)
White	36 (90.0)	112 (94.9)	381 (93.8)
Other	2 (5.0)	5 (4.2)	20 (4.9)
**Psoriasis disease duration, years**	17.6 ± 12.5	18.1 ± 11.6	18.2 ± 12.5
**Nx**	40	118	405
**Tobacco use**, ***n*** **(%)**			
Baseline at Week 0	5 (12.5)	42 (35.6)	154 (38.0)
**BMI, kg/m** ^**2**^	36.3 ± 8.1	32.6 ± 7.0	28.9 ± 6.5
Normal (≥18.5– <25), *n* (%)	2 (5.0)	9 (7.6)	105 (25.9)
Overweight (≥25– <30), *n* (%)	8 (20.0)	41 (34.7)	157 (38.7)
Obese (≥30– <40), *n* (%)	19 (47.5)	52 (44.1)	113 (27.8)
Extreme obese (≥40), *n* (%)	11 (27.5)	16 (13.6)	29 (7.1)
**Baseline Weight, kg**	107.4 ± 25.6	98.2 ± 21.8	86.7 ± 21.5
**Nx**	40	118	380
**Fasting serum glucose level, mmol/L**,	8.8 ± 3.8	6.4 ± 1.2	5.0 ± 0.4
**PASI**	20.7 ± 7.9	21.1 ± 7.7	19.9 ± 7.6
**BSA (%)**	31.2 ± 21.1	27.2 ± 16.1	27.4 ± 16.4
**sPGA**	3.5 ± 0.6	3.5 ± 0.6	3.5 ± 0.6
**DLQI, (Nx)**	13.8 ± 7.7	12.3 ± 6.8	(405) 12.1 ± 7.0
**Previous biologic use**, ***n*** **(%)**	12 (30.0)	26 (22.0)	84 (20.7)
**Psoriatic arthritis**, ***n*** **(%)**	8 (20.0)	30 (25.4)	83 (20.4)
**Hypertension**, ***n*** **(%)**	31 (77.5)	43 (36.4)	84 (20.7)

Nx for BMI and Fasting serum glucose level for patient group with normoglycemia differs from N. Hypertension and hyperlipidemia were defined as the proportion of patients with a pre-existing history of or test results indicative of hypertension and hyperlipidemia, respectively, at baseline. Data presented as mean ± standard deviation unless otherwise indicated. BMI, body mass index; BSA, Body Surface Area; DLQI, Dermatology Life Quality Index; *N*, number of patients in the analysis population; *n*, number of patients in the specified category; Nx, number of patients with non-missing values; PASI, Psoriasis Area and Severity Index; sPGA, Static Physician Global Assessment; T2D, type 2 diabetes.

### Efficacy outcomes

**Figure 1:**
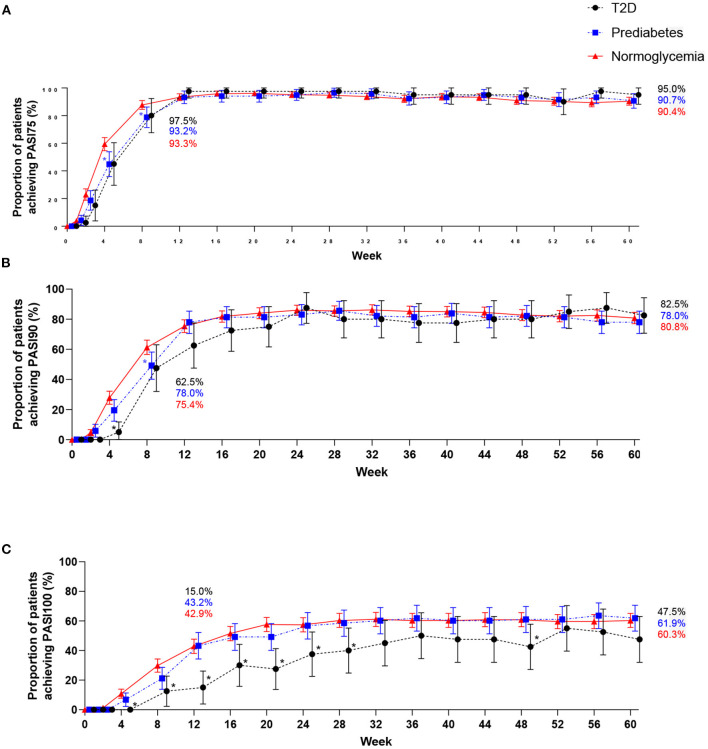
PASI outcomes through Week 60 (NRI). Percentage of patients with type 2 diabetes, prediabetes, and without type 2 diabetes or prediabetes (normoglycemia) achieving PASI75 **(A)**, PASI90 **(B)**, and PASI100 **(C)**. n, number; PASI, Psoriasis Area and Severity Index; T2D, type 2 diabetes. Symbols in line graphs are offset horizontally to differentiate overlapping data points. 95% confidence intervals are shown. * indicates *p* < 0.05, T2D vs. normoglycemia, * indicates *p* < 0.05, prediabetes vs. normoglycemia.

Similar proportions of patients across the three groups achieved PASI75 and PASI90 at W60, under comparable treatment duration between responses over the study period. Patients showed a rapid improvement at W1 (PASI75) and W4 (PASI90) of treatment and maintained improvements from W12 through to W60, ensuing a plateau in skin improvement (Figures 1A, B). In contrast, it appears that patients with T2D and to less extent with prediabetes had a slower onset for achieving PASI100 compared to patients with normoglycemia; however, at W60, all outcomes across the three groups were similar (Figure 1C).

### Clinical measures and metabolic markers

At baseline, fasting serum glucose concentrations were higher in patients with prediabetes and markedly elevated T2D compared with patients with normoglycemia (Table 2). Patients with T2D had lower levels of total serum cholesterol and low-density lipoprotein cholesterol compared with patients with prediabetes and normoglycemia. It is undetermined whether the lower levels recorded were due to patients receiving concomitant medication or not. All other clinical measures and metabolic markers were similar across patients with prediabetes, T2D, and normoglycemia at baseline.

**Table 2:** Clinical and metabolic markers at baseline, Week 12 and Week 60.

**Clinical and metabolic markers**	**T2D (*N =* 40) Nx (mean ±SD)**	**Prediabetes (*N =* 118) Nx (mean ±SD)**	**Normoglycemia (*N =* 406) Nx (mean ±SD)**
**BMI, kg/m** ^2^			
Baseline	36.3 ± 8.1	32.6 ± 7.0	28.9 ± 6.5
Week 60	36.1 ± 7.7	32.6 ± 6.9	29.0 ± 6.6
**Systolic blood pressure, mmHg**			
Baseline	133.0 ± 12.5	132.3 ± 12.5	125.4 ± 14.7
Week 12	132.9 ± 12.8	129.6 ± 12.6	125.9 ± 13.4
Week 60	128.8 ± 13.0	129.9 ± 12.8	124.2 ± 12.7
**Diastolic blood pressure, mmHg**			
Baseline	81.3 ± 8.3	80.4 ± 8.2	79.2 ± 10.0
Week 12	80.9 ± 8.5	80.6 ± 8.0	78.5 ± 9.4
Week 60	79.2 ± 10.8	81.0 ± 9.4	78.5 ± 9.5
**Fasting serum glucose level, mmol/L**			
Baseline	7.6 ± 2.1	6.1 ± 1.5	5.1 ± 0.6
Week 12	N/A	5.5 ± N/A	5.3 ± 0.6
Week 60	8.9 ± 3.0	6.1 ± 1.2	5.2 ± 0.7
**Serum cholesterol level, mmol/L**			
Baseline	4.5 ± 1.2	5.4 ± 1.1	5.2 ± 1.1
Week 12	N/A	1 (4.1 ± N/A)	9 (5. ± 1.1)
Week 60	38 (4.5 ± 1.0)	109 (5.4 ± 1.1)	359 (5.2 ± 0.1)
**LDL cholesterol level, mmol/L**			
Baseline	39 (2.5 ± 1.1)	117 (3.2 ± 1.0)	403 (3.1 ± 1.0)
Week 12	N/A	1 (2.2 ± N/A)	8 (3.1 ± 0.8)
Week 60	36 (2.5 ± 1.0)	105 (3.2 ± 1.0)	351 (3.1 ± 0.9)
**HDL cholesterol level, mmol/L**			
Baseline	1.2 ± 0.3	1.2 ± 0.4	1.4 ± 0.4
Week 12	N/A	1 (1.2 ± N/A)	9 (1.3 ± 0.4)
Week 60	38 (1.2 ± 0.3)	109 (1.2 ± 0.4)	357 (1.4 ± 0.4)
**VLDL cholesterol level, mmol/L**			
Baseline	39 (0.8 ± 0.4)	117 (0.9 ± 0.4)	403 (0.7 ± 0.4)
Week 12	N/A	1 (0.8 ± N/A)	8 (1.0 ± 0.5)
Week 60	0.8 ± 0.4	0.9 ± 0.4	0.7 ± 0.4
**Serum triglyceride level, mmol/L**			
Baseline	40 (1.8 ± 1.0)	118 (2.1 ± 1.1)	406 (1.7 ± 1.1)
Week 12	N/A	1 (1.7 ± N/A)	9 (2.5 ± 1.5)
Week 60	38 (2.0 ± 1.2)	109 (2.1 ± 1.0)	359 (1.7 ± 1.1)
**Apolipoprotein B level, g/L**			
Baseline	40 (1.4 ± 0.2)	116 (1.4 ± 0.3)	394 (1.5 ± 0.3)
Week 12	N/A	1 (1.4 ± N/A)	9 (1.4 ± 0.2)
Week 60	37 (0.9 ± 0.2)	108 (1.0 ± 0.3)	358 (1.0 ± 0.3)
**Apolipoprotein A1 level, g/L**			
Baseline	1.4 ± 0.2	116 (1.4 ± 0.3)	394 (1.5 ± 0.3)
Week 12	N/A	1 (1.4 ± N/A)	9 (1.4 ± 0.2)
Week 60	37 (1.4 ± 0.2)	108 (1.5 ± 0.2)	358 (1.5 ± 0.3)

Where no Nx value, Nx and N are the same. BMI, body mass index; HDL, high-density lipoprotein; LDL, low-density lipoprotein; *N*, number of patients in the analysis population; n, number of patients in the specified category; Nx, number of patients with non-missing values; SD, standard deviation; T2D, type 2 diabetes; VLDL, very-low-density lipoprotein.

Ixekizumab treatment had no effect on all metabolic markers mentioned in Table 2 after W12 and W60 of treatment. Importantly, clinical measures and metabolic markers did not worsen in ixekizumab-treated patients with prediabetes or T2D through to W60, despite the expected progression of glycemic impairment with time (13).

## Discussion

This study investigating the efficacy of ixekizumab in treating moderate-to-severe psoriasis in patients with concomitant prediabetes or T2D has revealed several important findings. Of note, comparable proportions of patients with prediabetes, T2D, and normoglycemia achieved PASI75, PASI90, and PASI100 at W60. All patients across the three groups achieved PASI75 and PASI90 at a similar response rate; however, patients with T2D achieved PASI100 more slowly than patients with prediabetes or normoglycemia. Results reported in other clinical studies indicate that the co-existence of metabolic syndrome rather than psoriasis disease severity could potentially impact the response to biological treatment (4, 8, 14). Obesity may be associated with reduced treatment responses to some biologics for psoriasis, possibly due to lower serum concentrations in obese patients where dose of the therapy is not weight-adjusted. For example, a pooled analysis of three phase 3 clinical trials evaluating the effect of bodyweight in response to ixekizumab treatment in patients with moderate-to-severe psoriasis, showed some numerical difference in PASI 90 and PASI 100 response rates between bodyweight categories (<80 kg, 80– <100 kg, and ≥100 kg) for ixekizumab and etanercept over a 12-week period (4, 15). As our patients with psoriasis and T2D had high rates of obesity and extreme obesity, which may have an effect on drug clearance, it is possible that this prevented some of them from achieving complete skin clearance and that weight reduction should also be included in the treatment plan (16). Furthermore, as T2D amplifies underlying pathophysiological inflammatory mechanisms of psoriasis, further investigation of the efficacy of ixekizumab in patients with metabolic syndrome is needed to further optimize the treatment on offer for patients with T2D.

The clinical measures and metabolic markers in ixekizumab-treated patients with prediabetes or T2D did not worsen through W60, despite the progressive nature of these diseases.

This study has some limitations. Given the *post-hoc* and descriptive nature of these analysis, low patient numbers potentially resulting in a low precision of the studied effect estimates, and high prevalence of other metabolic disorders in patients with prediabetes and T2D, caution should be used when interpreting these results as statistical comparisons could not be made.

## Conclusion

In summary, this *post-hoc* analysis demonstrated that ixekizumab is efficacious in patients with psoriasis and prediabetes or T2D, despite high rates of obesity and extreme obesity in this group. Our results show that similar proportions of patients with prediabetes, T2D, and normoglycemia achieve PASI75, PASI90, and PASI100 at a comparable rate; however, patients with T2D may take longer to achieve PASI100. Our findings suggest that patients with psoriasis and concomitant prediabetes or T2D achieve a comparable response to treatment in the context of psoriasis compared to those with normoglycemia. This study provides clinicians with the evidence that while patients with T2D may take longer to respond, ixekizumab is effective for the treatment of patients with moderate-to-severe psoriasis who have comorbid prediabetes or T2D.

## Data availability statement

The data analyzed in this study is subject to the following licenses/restrictions: Lilly provides access to all individual participant data collected during the trial, after anonymization, with the exception of pharmacokinetic or genetic data. Data are available to request 6 months after the indication studied has been approved in the US and EU and after primary publication acceptance, whichever is later. No expiration date of data requests is currently set once data are made available. Access is provided after a proposal has been approved by an independent review committee identified for this purpose and after receipt of a signed data sharing agreement. Data and documents, including the study protocol, statistical analysis plan, clinical study report, blank or annotated case report forms, will be provided in a secure data sharing environment. For details on submitting a request, see the instructions provided at www.vivli.org. Requests to access these datasets should be directed to www.vivli.org.

## Ethics statement

All patients provided written informed consent prior to enrollment. All studies were conducted with the approval of each Center's Institutional Review Board or Independent Ethics Committee and in accordance with the guiding principles of the Declaration of Helsinki. The patients/participants provided their written informed consent to participate in this study.

## Author contributions

PM, CS, and IP contributed to conception and design of the study. IW performed the statistical analysis. All authors contributed to manuscript revision, read, and approved the submitted version.
