# Corrigendum: Efficacy of ixekizumab in patients with moderate-to-severe plaque psoriasis and prediabetes or type 2 diabetes

**DOI:** 10.3389/fmed.2023.1171132

**Published:** 2023-03-10

**Authors:** Alexander Egeberg, Joseph F. Merola, Knut Schäkel, Luis Puig, Patrick D. Mahar, Isabella Yali Wang, Imre Pavo, Christopher Schuster, Christopher E. M. Griffiths

**Affiliations:** ^1^Bispebjerg and Frederiksberg Hospital, Copenhagen University, Copenhagen, Denmark; ^2^Division of Rheumatology, Department of Dermatology and Department of Medicine, Harvard Medical School, Brigham and Women's Hospital, Boston, MA, United States; ^3^Department of Dermatology, University Hospital Heidelberg, Heidelberg, Germany; ^4^Dermatology Department, Hospital de la Santa Creu i Sant Pau, Barcelona, Spain; ^5^Eli Lilly and Company, Indianapolis, IN, United States; ^6^Department of Dermatology, Royal Children's Hospital, Faculty of Medicine, Nursing and Health Sciences, Skin Health Institute, The University of Melbourne, Melbourne, VIC, Australia; ^7^Department of Dermatology, Medical University of Vienna, Vienna, Austria; ^8^Dermatology Centre, Salford Royal Hospital, NIHR Manchester Biomedical Research Centre, University of Manchester, Manchester, United Kingdom

**Keywords:** moderate-to-severe psoriasis, type 2 diabetes, prediabetes, ixekizumab, obesity

In the published article, there was an error regarding the Conflict of Interest for Patrick D. Mahar, Christopher Schuster, Imre Pavo, and Isabella Yali Wang. The Conflict of Interest statement should be:

AE has honoraria as consultant and/or speaker from AbbVie, Almirall, Bristol-Meyers Squibb, Leo Pharma, Samsung Bioepis Co., Ltd., Pfizer, Eli Lilly, Novartis, Galderma, and Janssen Pharmaceuticals. LP has received honoraria/consultation fees from Abbvie, Almirall, Amgen, Baxalta, Boehringer Inglheim, Celgene, Gebro, Janssen, Leo-Pharma, Lilly, Merck-Serono, MSD, Mylan, Norvartis, Pfizer, Regeneron, Roche, Sandoz, Samsung-Bioepis, Sanofi and UCB. LP has participated in company sponsored speaker's bureau for Celgene, Janssen, Lilly, MSD, Norvartis and Pfizer. KS is a consultant, investigator, speaker and has received grants from: AbbVie, Amgen, Almirall, Biogen, Bristol-Myers Squibb Boehringer Ingelheim, Celgene, Chugai, Galderma, Janssen-Cilag, Leo-Pharma, Lilly, Merck Sharp & Dohme Corp., Morphosys, Novartis, Pfizer, Regeneron, UCB Pharma. JM is a consultant and/or investigator for Amgen, Bristol-Myers Squibb, Abbvie, Dermavant, Eli Lilly, Novartis, Janssen, UCB, Sanofi-Regeneron, Sun Pharma, Biogen, Pfizer and Leo Pharma. CG has received honoraria or research grants from AbbVie, Almirall, Anaptysbio Boehringer Ingelheim, Bristol Myers Squibb, Eli Lilly and Company, GSK, Janssen, LEO Pharma, Pfizer, Novartis ONO Pharmaceuticals, UCB Pharma and Walgreens Boots Alliance, and is supported in part by the Manchester NIHR Biomedical Research Centre. PM has served as a consultant, investigator, speaker and/or advisor for Novartis, AstraZeneca, Abbvie, Pfizer, Bristol-Meyers Squibb, Eli Lilly and Company and Boehringer Ingelheim. PM, CS, IP, and IW are employees of Eli Lilly and Company. PM owns equity in Eli Lilly and Company. CS, IP, and IW are minor shareholders of Eli Lilly and Company.

The authors apologize for this error and state that this does not change the scientific conclusions of the article in any way. The original article has been updated.

